# Skeletal muscle wasting and long-term prognosis in patients undergoing rectal cancer surgery without neoadjuvant therapy

**DOI:** 10.1186/s12957-021-02460-7

**Published:** 2022-02-25

**Authors:** Alessandro Giani, Simone Famularo, Alessandro Fogliati, Luca Riva, Nicolò Tamini, Davide Ippolito, Luca Nespoli, Marco Braga, Luca Gianotti

**Affiliations:** 1 Present Address: School of Medicine and Surgery, University of Milano-Bicocca, Monza, Italy; 2 Department of Surgery, San Gerardo Hospital, Monza, Italy; 3 Department of Radiology, San Gerardo Hospital Via Pergolesi 33, 20900 Monza, Italy

**Keywords:** Sarcopenia, Body composition, Rectal cancer, Surgery, Long-term survival

## Abstract

**Background:**

Derangement of body composition has been associated with dismal long-term survival in several gastrointestinal cancers including rectal tumors treated with neoadjuvant therapies. The role of specific preoperative anthropometric indexes on the oncologic outcomes of patients undergoing upfront surgery for rectal cancer has not been investigated.

The aim of the study is to evaluate the association of body composition and overall survival in this specific cohort.

**Methods:**

Lumbar computed tomography images, obtained within the 30 days previous to surgery, between January 2009 and December 2016, were used to calculate population-specific thresholds of muscle mass (sarcopenia), subcutaneous and visceral adiposity, visceral obesity, sarcopenic obesity, and myosteatosis. These body composition variables were related with overall survival (OS), tumor-specific survival (TSS), and disease-free survival (DFS). OS, TSS, and DFS were evaluated by the Kaplan-Meier method. Cox regression analysis was used to identify independent predictors of mortality, tumor-specific mortality, and recurrence, and data were presented as hazard ratio (HR) and 95% confidence interval (CI).

**Results:**

During the study period, 411 patients underwent rectal resection for cancer, and among these, 129 were without neoadjuvant chemoradiation. The median follow-up was 96.7 months. At the end of the follow-up, 41 patients (31.8%) had died; of these, 26 (20.1%) died for tumor-related reasons, and 36 (27.1%) experienced disease recurrence.

One-, three-, and five-year OS was 95.7%, 86.0%, and 76.8% for non-sarcopenic patients versus 82.4%, 58.8%, and 40.0% for sarcopenic ones respectively (*p* < 0.001). Kaplan-Meier survival curves comparing sarcopenic and non-sarcopenic patients showed a significant difference in terms of OS (log-rank < 0.0001). Through multivariate Cox regression, overall mortality risk was associated only with sarcopenia (HR 1.96; 95%CI 1.03–3.74; *p* = 0.041). Disease stage IV and III (HR 13.75; 95% CI 2.89–65.6; *p* < 0.001 and HR 4.72; 95% CI 1.06–21.1; *p* = 0.043, respectively) and sarcopenia (HR 2.62; 95% CI 1.22–5.6; *p* = 0.013) were independently associated with TSS. The other body composition indexes investigated showed no significant association with prognosis.

**Conclusions:**

These results support the inclusion of body composition assessment for prognostic stratification of rectal cancer patients undergoing upfront resection.

**Supplementary Information:**

The online version contains supplementary material available at 10.1186/s12957-021-02460-7.

## Introduction

In recent years, the impact of anthropometry on outcome is a matter of extensive research in several fields of surgery [1, 2]. Cancer-bearing patients are routinely staged before operation by way of computed tomography (CT) scan that also allows the estimation of quantitative and qualitative measurements of body composition [3, 4]. The assessment of body structure through CT scan is used to stratify the risk of short-term postoperative morbidity and mortality in patients undergoing surgical procedures for malignant disease of the lung [5], liver [6], stomach [7], pancreas [8], and colorectum [9]. Likewise, specific body composition indexes seem to be associated with dismal long-term prognosis of patients suffering pancreatic, gastric, pulmonary, breast, biliary, and urinary tract tumors [5, 7, 10–13]. Recently, some studies have apprised the association of sarcopenia, visceral adiposity, and myosteatosis with worse overall and disease-free survival in patients undergoing colorectal surgery for cancer [14–17]. In those reports, colon and rectal cancers were evaluated as a single entity despite the fact that their perioperative management and treatment strategies are substantially different. Rectal surgery accounts for a substantially greater risk of postoperative complications and, in particular, higher rates of anastomotic leakage [18, 19] and surgical site infection [20] than those reported for colon resections. Moreover, rectal tumors appear to have a worse long-term prognosis than colonic ones [21–24]. Accordingly, it would be worthwhile to study more homogeneous patient populations. Neoadjuvant chemoradiation (NACR) should be the standard of care for stage II–III rectal cancer [25] given its advantages in terms of survival and recurrence. However, NACR is not indicated in the early and metastatic disease stages and its role is still debated in intermediate stage disease when a good quality mesorectal excision can be achieved [25–27]. Additionally, patients may not undergo NACR due to poor performance status or personal preference. Furthermore, neoadjuvant treatments may affect body composition [28] potentially resulting a confounding factor.

The aim of the present study is to evaluate the potential association of preoperative body composition indexes and long-term oncologic outcomes in a cohort of patients undergoing rectal resection for cancer without NACR.

## Materials and methods

### Study overview

A prospectively maintained and anonymized electronic dataset was queried for adult patients who had undergone curative surgery for rectal cancer between January 2009 and December 2016 at our institution. The exclusion criteria were the lack of a CT scan performed locally within 30 days previous to surgery and that they were receiving NACR. For data analysis, the oncologic follow-up was interrupted in May 2020. The local ethical committee review of the protocol deemed that formal approval was not required owing to the retrospective, observational, and anonymous nature of this study. The study protocol followed the ethical guidelines of the 1975 Declaration of Helsinki (revised in Brazil in 2013). Results are reported in accordance with Strengthening the Reporting of Observational Studies in Epidemiology (STROBE) [29].

### CT-derived body composition analysis

The last CT scan performed before surgical resection was used for the analysis. A multiphasic multidetector CT scan was performed with two different CT scanners: The Brilliance iCT 256-slice or Brilliance 16-slice CT scanners (Philips Medical Systems, Eindhoven, Netherlands). An unenhanced scan was followed by a post-contrast triphasic acquisition (arterial, portal venous, and equilibrium phase), after the intravenous injection of 90 to 145 mL of non-ionic iodinated contrast medium (Xenetix 350; Guerbet, Aulnay, France) at a flow-rate of 3.5 mL/s followed by the injection of 50 mL of saline solution. Images were reconstructed with the Filtered Back projection technique if acquired on Brilliance 16-slice CT or with the Hybrid-Iterative Reconstruction algorithm (iDose4) if acquired on Brilliance iCT. All the examinations were transferred to an image workstation (Intellispace portal 8.0; Philips Medical Systems) to evaluate, select, and save the image for analysis, in DICOM format. The analysis was performed with the open source image analysis software ImageJ (developed by the National Institutes of Health; available from http://rsbweb.nih.gov/ij/download.html), which gives comparable results of other soft-ware for body composition analysis, as previously described by van Vugt et al. [3].

Two dedicated radiologists (LR, DI), blinded to patient information, calculated total muscle area (TMA), which estimates the total muscle mass, total fat area (TFA), visceral fat area (VFA), subcutaneous fat area (SFA), and intramuscular fat area (IMFA) [4]. TMA, SFA and VFA were utilized to calculate the skeletal muscle index (SMI = TMA/m^2^), subcutaneous adipose tissue index (SATI = SFA/m^2^), and sarcopenic obesity (SO=VFA/TMA). The visceral to subcutaneous adipose tissue area ratio was calculated to explore the abdominal adipose tissue distribution (VSR = VFA/SFA). The grade of myosteatosis was determined through the intramuscular adipose tissue content (MS = IMFA/TMA). Body composition indexes were normalized for height in meters squared [3, 30] and expressed as cm^2^/m^2^. The first and the fourth quartile were estimated for each index according to sex [31]. Body composition indexes were presented as dichotomous variables. The first quartile of SMI was considered to be sarcopenia, while the last quartile was used for VATI, SATI, VSR, VFMAI, and IMFAR to quantify visceral adiposity, high subcutaneous adiposity, visceral obesity (VO), sarcopenic obesity (SO), and myosteatosis, respectively. Patients were considered obese when BMI > 30 kg/m^2^ and underweight when BMI < 20 kg/m^2^ [30].

### Outcome variables and definitions

Age, sex, the Charlson comorbidity index (CCI), and the American Society of Anesthesiology (ASA) score were collected during the first outpatient visit. The type of operation (anterior resection, Miles procedure or Hartmann procedure) and approach (open or laparoscopic) were the surgical parameters considered. Histology variables were evaluated and included infiltration of resection margins (R status), pTNM, and disease stage. The Clavien-Dindo Classification was adopted to stratify postoperative morbidity and mortality [32]. The follow-up data included the date of death, the date of cancer relapse, and the site of relapse and were collected according to the latest outpatient visit. When oncologic follow-up was not available in the hospital registry, patients were reached by telephone interviews. Overall survival (OS) was defined as the time interval in months from surgery to death; if the patient was alive, data were censored at the last available visit. Tumor-specific survival (TSS) was defined as the time interval in months from surgery to cancer-related death. Other specific causes of death were not recorded, but when occurred, patients were censored. Disease-free survival (DFS) was defined as the time interval in months from surgery to recurrence or death. In case of no recurrence, data were censored at the date of the last available follow-up. Patients with stage IV cancer were excluded from this latter analysis.

### Study endpoints

The primary endpoint was to evaluate the possible association of different body composition indexes and OS in patients undergoing rectal resection for cancer who did not receive NACR. Secondary endpoints were the differences in terms of DFS and TSS among different anthropometric phenotypes.

### Statistics

Data are expressed as a median and interquartile range (IQR) and number and relative percentage. Normal distribution of continuous variables was assessed using the Kolmogorov-Smirnov test. Continuous variables were analyzed using the Mann-Whitney test and categorical variables using the Fisher exact test or chi-square test as appropriate. OS, TSS, and DFS were evaluated using the Kaplan-Meier method. Stage IV patients were excluded from the DFS analysis. Comparison among groups was performed using the log-rank test. Cox regression analysis was used to identify independent predictors of mortality, tumor-specific mortality, and recurrence, and data was presented as hazard ratio (HR) and 95% confidence interval (CI). Variables to be inserted in the model were selected by a step-forward approach with all the significant (*p* < 0.1) and clinically relevant variables examined by univariate Cox analysis. Median follow-up time was estimated with the reverse Kaplan-Meier method. All statistics were 2-tailed, and statistical significance was accepted when *p* < 0.05. All statistical analyses were performed using R software (v 3.6.0).

## Results

A total of 411 patients underwent a rectal resection for cancer during the study period. Among them, 129 patients did not receive NACR and thus were included in the analysis. The reasons they did not receive NACR were as follows: 13 patients were stage I, 30 were stage IV, 64 due to the upper tumor limit crossing over the peritoneal reflection, 13 due to comorbidities, and 9 due to refusal.

**Fig. 1:**
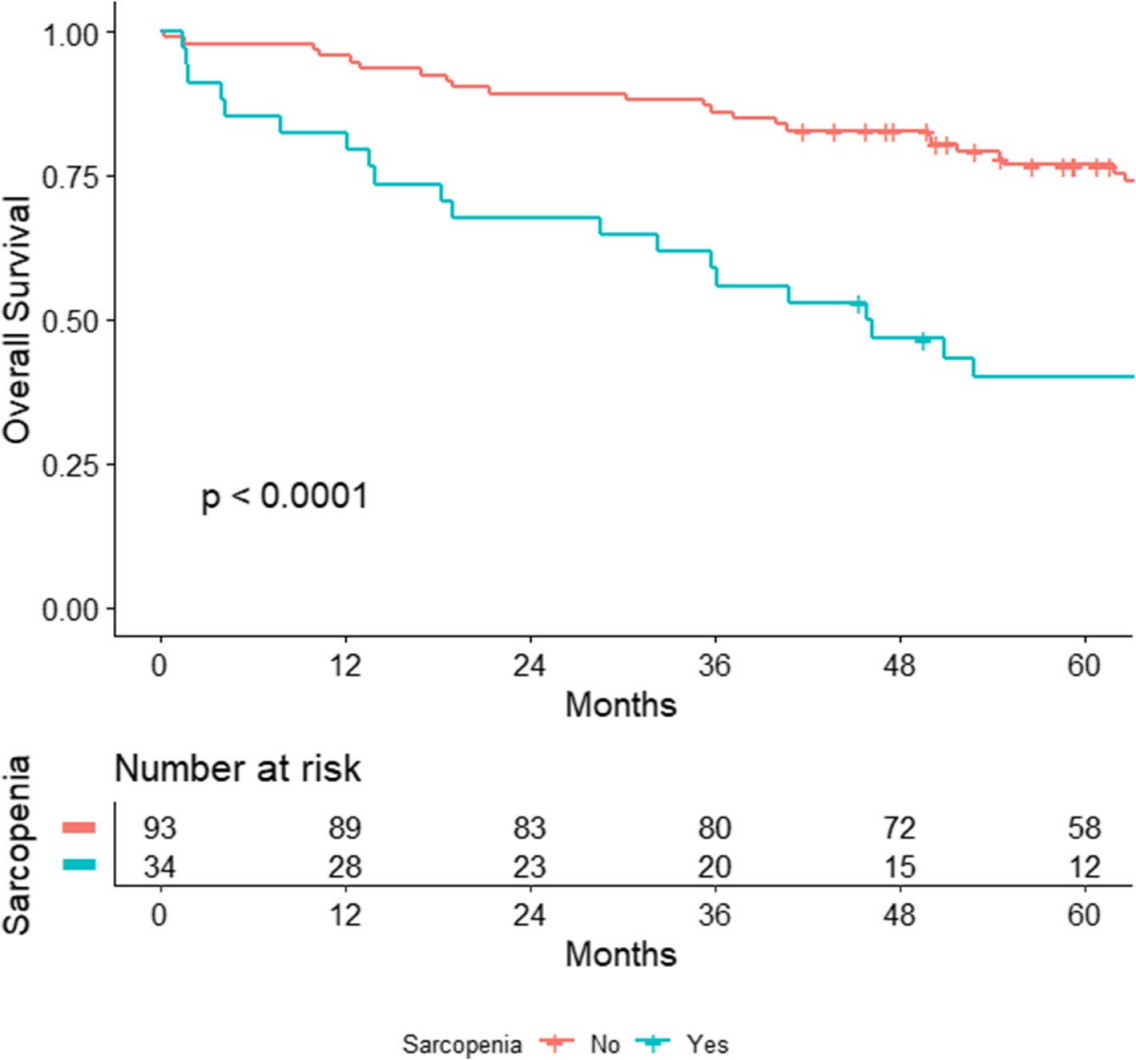
Kaplan-Meier overall survival curves for sarcopenic and non sarcopenic rectal cancer patients

Baseline, surgical, and tumor characteristics are shown in Table 1. Briefly, the median age of the cohort was 72 years (IQR 62–78), and 48 (37.2%) were females. The median CCI was 5 (IQR 4–7), the median body mass index (BMI) 25.3 (IQR 23.4–27.8), and laparoscopic resection was performed for 62 patients (48.1%). Body composition indexes, their relative ranges, and the cutoff values used for the present analysis are reported in Supplementary Table 1. The median follow-up was 96.7 months (95% CI 61.6–119.5). At the end of the follow-up, overall, 41 patients (31.8%) had died; of these, 26 (20.1%) for tumor-related reasons. Thirty-five patients (27.1%) experienced disease recurrence. Table 2 reports the Cox univariate analysis performed to identify factors predicting OS, TSS, and recurrence. The variables significantly associated with OS were age (HR 1.06, *p* < 0.001), CCI (HR 1.36, *p* < 0.001), BMI (HR 0.88, *p* = 0.006), SMI (HR 3.1, *p* < 0.001), positive resection margins (HR 4.97, *p* < 0.001), VATI (HR 0.36, *p* = 0.021), and stage IV disease (HR 4.32, *p* = 0.003). TSS resulted correlated with CCI (HR 1.31, *p* = 0.001), BMI (HR 0.88, *p* = 0.025), SMI (HR 2.99, *p* = 0.004), VATI (HR 0.18, *p* = 0.021), positive resection margins (HR 7.96, *p* < 0.001), TNM stage III (HR 5.53, *p* = 0.024), TNM stage IV (HR 14.96, *p* = 0.001), and adjuvant chemotherapy (HR 2.62, *p* = 0.028). DFS was significantly associated with CCI (HR 1.23, *p* = 0.039) and BMI (HR 0.88, *p* = 0.046). At the multivariate Cox regression, overall mortality risk was associated with sarcopenia (HR 1.96; 95% CI 1.03–3.74; *p* = 0.041) (Table 3). Disease stage IV and III (HR 13.75; 95% CI 2.89–65.6; *p* < 0.001 and HR 4.72; 95% CI 1.06–21.1; *p* = 0.043, respectively) and sarcopenia (HR 2.62; 95% CI 1.22–5.6; *p* = 0.013) were independently associated with TSS (Supplementary Fig. 1). One-, three-, and five-year OS was 95.7%, 86.0%, and 76.8% for non-sarcopenic patients versus 82.4%, 58.8%, and 40.0% for the sarcopenic group respectively (*p* < 0.001). TSS at 1, 3, and 5 years was 96.8%, 90.2%, and 85.4% for non-sarcopenic patients versus 85.3%, 75.3%, and 54.7% (*p* = 0.002) in the case of sarcopenia respectively. DFS rates at 1, 3, and 5 years were 89.4%, 80.3%, and 70.6% for the non-sarcopenic group and 69.2%, 46.2%, and 46.2% in the case of sarcopenia respectively (*p* = 0.004). The Kaplan-Meier survival curve for OS is depicted in Fig. 1 while TSS and DFS curves are shown in Supplementary Figs. 2 and 3 respectively.

**Table 1:** Characteristics of the cohort

Variables		Included patients ***N*** = 129
		Median (IQR)/*N* (%)
**Age**	Years	72 (62–78)
**Sex**	Females	48 (37.2)
**BMI**		25.3 (23.4–27.8)
**CCI**		5 (4–7)
**ASA score**	1	4 (3.1)
	2	66 (51.2)
	3	59 (45.7)
**Type of surgery**	Anterior resection	94 (72.9)
	Miles procedure	21 (16.3)
	Hartmann procedure	14 (10.8)
**Laparoscopy**		63 (48.8)
**Overall morbidity**		43 (33.3)
**Clavien-Dindo classification**	< 3	27 (20.9)
	≥ 3	15 (11.6)
**Redo surgery**		8 (6.2)
**Anastomotic leakage**^a^		8/94 (8.5)
**Tumor location**	High	64 (49.6)
	Middle	41 (31.8)
	Low	24 (18.6)
**TNM stage**	I	30 (23.3)
	II	33 (25.6)
	III	41 (31.8)
	IV	13 (10.1)
**Positive resection margin**		11 (8.5)
**Adjuvant chemotherapy**		61 (47.3)

*BMI* body mass index, *CCI* Charlson comorbidity index, *ASA* American Society of Anesthesiologists

^a^Calculated on the number of patients who underwent restorative surgery

**Table 2:** Values are hazard ration (HR) [95% confidential intervals]. Stage IV patients were excluded from analysis of disease-free survival

	Overall survival	Disease-free survival	Tumor-specific survival
Age	1.06 (1.03–1.09, *p* < 0.001)	1.03 (0.99–1.07, *p* = 0.137)	1.02 (0.99–1.06, *p* = 0.211)
Sex, female	0.79 (0.43–1.44, *p* = 0.444)	0.67 (0.27–1.64, *p* = 0.376)	0.69 (0.32–1.52, *p* = 0.360)
BMI	0.88 (0.80–0.96, *p* = 0.006)	0.88 (0.77–1.00, *p* = 0.046)	0.88 (0.78–0.98, *p* = 0.025)
CCI	1.36 (1.20–1.53, *p* < 0.001)	1.23 (1.01–1.50, *p* = 0.039)	1.31 (1.12–1.53, *p* = 0.001)
ASA score			
2 vs 1	0.45 (0.10–1.97, *p* = 0.291)	0.95 (0.13–7.14, *p* = 0.957)	0.73 (0.09–5.57, *p* = 0.758)
3 vs 1	1.21 (0.29–5.07, *p* = 0.796)	1.43 (0.19–10.93, *p* = 0.731)	1.22 (0.16–9.22, *p* = 0.850)
SMI, yes	3.15 (1.76–5.65, *p* < 0.001)	2.25 (0.93–5.45, *p* = 0.073)	2.99 (1.43–6.23, *p* = 0.004)
VO, yes	1.02 (0.54–1.94, *p* = 0.947)	1.97 (0.84–4.61, *p* = 0.118)	0.85 (0.36–1.98, *p* = 0.703)
IMFAR, yes	1.28 (0.69–2.40, *p* = 0.435)	0.71 (0.26–1.92, *p* = 0.495)	0.78 (0.32–1.92, *p* = 0.590)
SO, yes	0.72 (0.36–1.46, *p* = 0.367)	1.36 (0.55–3.33, *p* = 0.506)	0.31 (0.09–1.02, *p* = 0.054)
SATI, yes	0.78 (0.38–1.61, *p* = 0.503)	1.31 (0.51–3.35, *p* = 0.575)	0.52 (0.18–1.51, *p* = 0.231)
VATI, yes	0.36 (0.15–0.86, *p* = 0.021)	0.74 (0.27–2.01, *p* = 0.560)	0.18 (0.04–0.77, *p* = 0.021)
Laparoscopy, yes	0.49 (0.07–3.62, *p* = 0.485)	0.11 (0.01–0.89, *p* = 0.039)	0.36 (0.05–2.67, *p* = 0.315)
Severe complication, yes	0.84 (0.32–2.18, *p* = 0.717)	0.16 (0.02–1.24, *p* = 0.079)	0.85 (0.26–2.77, *p* = 0.792)
Tumor location			
Middle vs high	1.28 (0.67–2.46, *p* = 0.454)	0.67 (0.23–1.93, *p* = 0.461)	1.18 (0.50–2.75, *p* = 0.710)
Low vs high	1.37 (0.63–2.98, *p* = 0.434)	1.64 (0.57–4.72, *p* = 0.362)	1.46 (0.56–3.85, *p* = 0.441)
TNM stage			
II vs I	1.63 (0.68–3.94, *p* = 0.277)	1.23 (0.33–4.58, *p* = 0.759)	3.00 (0.61–14.87, *p* = 0.179)
III vs I	1.68 (0.72–3.93, *p* = 0.232)	2.88 (0.94–8.84, *p* = 0.064)	5.53 (1.25–24.51, *p* = 0.024)
IV vs I	4.32 (1.65–11.27, *p* = 0.003)	–	14.96 (3.16–70.84, *p* = 0.001)
Radicality			
R1 vs R0	4.97 (2.27–10.87, *p* < 0.001)	–	7.96 (3.27–19.39, *p* < 0.001)
Adjuvant chemotherapy, yes	1.19 (0.66–2.17, *p* = 0.563)	2.36 (0.92–6.04, *p* = 0.072)	2.62 (1.11–6.19, *p* = 0.028)

*BMI* Body mass index, *CCI* Charlson comorbidity index, *ASA* American Society of Anesthesiologists, *SMI* Skeletal muscle index, *VATI *Visceral adipose tissue index, *SATI* Subcutaneous adipose tissue index, *VO* Visceral obesity, *SO* Sarcopenic obesity

**Table 3:** Multivariate analysis of factors associated with overall mortality

	HR	95%CI	p
Age (per year of increase)	1.02	0.98-1.10	0.398
BMI (per point of increase)	0.92	0.83-1.0	0.107
CCI (per point of increase)	1.17	0.96-1.40	0.124
Sarcopenia (vs normal)	1.96	1.03-3.7	0.041
Stage II (vs I)	1.14	0.47-2.80	0.775
Stage III (vs I)	1.29	0.54-3.10	0.573
Stage IV (vs I)	2.46	0.82-7.40	0.107
VATI (low vs high)	0.65	0.26-1.60	0.347

*CI* Confidential interval, *BMI* Body mass index, *CCI* Charlson comorbidity index, *VATI* Visceral adipose tissue index

## Discussion

In this cohort study, regarding patients who underwent upfront rectal cancer surgery without NACR, the presence of preoperative sarcopenia assessed at CT scan was an independent prognostic factor for dismal long-term OS and TSS, along with other acknowledged variables such as the tumor stage. Several other studies have reported an association between preoperative skeletal muscle wasting and poor long-term survival in different oncological diseases [5–8, 33] and more specifically in colorectal cancer patients [15, 34–38]. However, all those trials used a combined analysis of colon and rectal cancer patients and this may have generated an ambiguous interpretation regarding the oncologic prognosis since colonic tumors have a better survival than rectal cancers [21–23, 39]. Only more recently have a few authors analyzed patients with rectal cancer alone [40, 41]. However, in these previous studies, all patients had received NACR. Since NACR may affect body composition variables [28], we decided to evaluate a specific cohort of patients undergoing rectal resection without having received NACR. In this context, the present data may add value to the previous literature since this group of patients had not been previously investigated. However, even in this relatively small and selected population, preoperative muscle wasting was significantly associated with dismal long-term prognosis as in other studies [40, 41].

Other authors have evaluated the role of visceral obesity and found no significant difference in the DFS and OS between the group bearing this condition and controls [16, 40, 42]. Similar to these findings, we did not observe a significant association of adiposity-related indexes, namely visceral obesity, sarcopenic obesity and myosteatosis, and survival. In contrast, Brown et al. [17] recently demonstrated the role of visceral and subcutaneous adiposity in affecting mortality in a large cohort of North American colorectal cancer patients. Instead, Han et al. [43] showed in a Korean population, that sarcopenic obesity was the only anthropometric variable negatively associated with overall survival in a specific subgroup of non-metastatic rectal cancer patients. In another recent study, Horii et al. found a negative impact of high intramuscular adipose tissue content on short and long-term outcomes in a subgroup of patients with colorectal liver metastasis undergoing hepatectomy [44]. This inconsistency of results may be partially explained by the different ethnicity and the well-recognized differences in anthropometric phenotype among Mediterranean, North American, and Asian populations.

The exact mechanism of how changes in body composition affect survival of cancer patients remains speculative. Sarcopenia is a component of cancer cachexia and a hallmark of malnutrition, and thus, it may be considered an indirect sign of aggressive disease and subsequent poor prognosis [45]. Nutritional risk scores are widely used and have been repeatedly demonstrated to be predictors of all-cause mortality and cancer-specific death in colorectal cancer [46, 47]. However, some authors have underlined how the quantitative measurement of muscle wasting overtakes classic malnutrition metrics as a predictor of survival [36, 45], and the recently proposed GLIM criteria for malnutrition now include instrumental measurement of the muscle mass [48].

Different pathophysiologic pathways have been proposed to explain the association between muscle mass or other body compartments and long-term survival. A systemic inflammation status seems a key mechanism [49]. Chronic inflammation increases the risk of cancer and hampers patient response to treatments [50]. High preoperative inflammatory markers have been directly associated to an increase in morbidity and mortality in colorectal cancer patients undergoing surgery [51]. Adipose tissue and skeletal muscle are the largest organs of the body and their immunoregulatory role are well recognized, given the ability to release a wide range of bioactive mediators [52]. In particular, myokines, cytokines produced by myocytes, play a critical role in cancer prevention counterbalancing the harmful effects of proinflammatory adipokines [52, 53].

The qualitative and quantitative evaluation of body architecture based on CT scan has several advantages. It does not add risk to patients since this imaging tool is routinely used for staging purposes, and thanks to new software, the measurement of different body compartments can be easily gathered. Furthermore, accuracy and reproducibility [3, 40] are additional reasons to propose abdominal CT imaging as the reference technique for anthropometric evaluation. These characteristics, combined with the prognostic ability, suggest adding CT-derived body composition assessment to conventional evaluation tools to identify individuals who might benefit from early and tailored metabolic interventions. By moving from measurement to action, multimodal prehabilitation programs, combining physical exercise and personalized nutritional intervention, in candidates to colorectal operations, have been recently shown to increase lean body mass and reduce fat mass [54]. Whether these changes in body composition will affect long-term outcomes of surgical cancer patients need to be further explored.

There are some limitations in our study. First, it was a single-center retrospective study and results need to be validated by prospective observational trials. Second, we used sex-related percentiles of our population rather than validated cutoffs to define groups at risk. Yet, body composition may have profound regional and ethnic variations, and peculiar diseases may have different influences on anthropometric changes, emphasizing the risk of using universal thresholds. Moreover, CT scans were obtained at the time of diagnosis, and the trajectory and overtime changes were not investigated. Lastly, there was a relatively small number of patients who experienced recurrence and cancer-related death, with a potential generation of a type-II error.

## Conclusions

In this cohort study of patients who underwent rectal cancer surgery without NACR, the presence of preoperative sarcopenia assessed at CT scan was an independent prognostic factor for dismal long-term survival. The present results support the inclusion of body composition assessment for prognostic stratification also for this cohort of patients.

## Supplementary Information

**Additional file 1: Supplementary Table 1** Descriptive analysis of the anthropometric measures and body composition indexes. Legend: Numbers are medians (percentile). BMI: Body mass index; SMI: Skeletal muscle index; VATI: Visceral adipose tissue index; SATI: Subcutaneous adipose tissue index; VFMAI: Visceral fat-muscle area index; VSR: Visceral to subcutaneous adipose tissue area ratio; IMFAR: Infra-muscular fat area ratio.

**Additional file 2: Supplementary Fig. 1** Multivariate analysis of factors associated to cancer-related death in rectal cancer patients undergoing upfront surgery. Quadrangles represent hazard risk. Horizontal bars represent 95% confidential interval VATI: Visceral adipose tissue index.

**Additional file 3: Supplementary Fig. 2** Kaplan-Meier tumor-specific survival curve for sarcopenic and non sarcopenic rectal cancer patients.

**Additional file 4: Supplementary Fig. 3** Kaplan-Meier disease-free survival curve for sarcopenic and non sarcopenic rectal cancer patients.

## Data Availability

The datasets analyzed during the current study are available from the corresponding author on reasonable request.
