# Supplementary material for: Skeletal muscle wasting and long-term prognosis in patients undergoing rectal cancer surgery without neoadjuvant therapy
Source: World J Surg Oncol. 2022 Feb 25;20:51. doi: 10.1186/s12957-021-02460-7 (PMC8881874; doi:10.1186/s12957-021-02460-7)
Supplement: Supplementary file 1 — Additional file 1: Supplementary Table 1 Descriptive analysis of the anthropometric measures and body composition indexes. Legend: Numbers are medians (percentile). BMI: Body mass index; SMI: Skeletal muscle index; VATI: Visceral adipose tissue index; SATI: Subcutaneous adipose tissue index; VFMAI: Visceral fat-muscle area index; VSR: Visceral to subcutaneous adipose tissue area ratio; IMFAR: Infra-muscular fat area ratio. [file 12957_2021_2460_MOESM1_ESM.docx]

**Supplementary Table 1.** Descriptive analysis of the anthropometric measures and body composition indexes.

|  | **Female (n=62)** | **Male (n=111)** |
| --- | --- | --- |
| BMI (kg/m^2^) | 25.7 (23.5-27.9) | 25.1 (23.5-27.5) |
| SMI (cm^2^/m^2^) | 38.7 (33.1-45.5) | 45.9 (38.2-51.9) |
| VATI (cm^2^/m^2^) | 53.8 (31.8-75.1) | 62.9 (40.8-86.1) |
| SATI (cm^2^/m^2^) | 80.9 (48.5-109.1) | 49.8 (36-59.3) |
| VFMAI (cm^2^/m^2^) | 1.3 (0.9-1.9) | 1.4 (1-1.8) |
| VSR | 0.6 (0.4-1) | 1.4 (0.9-1.6) |
| IMFAR | 0.29 (0.17-0.37) | 0.19 (0.14-0.28) |

Numbers are medians (percentile). BMI: Body mass index; SMI: Skeletal muscle index; VATI: Visceral adipose tissue index; SATI: Subcutaneous adipose tissue index; VFMAI: Visceral fat-muscle area index; VSR: Visceral to subcutaneous adipose tissue area ratio; IMFAR: Infra-muscular fat area ratio.
