# Supplementary figures and images for: Skeletal muscle wasting and long-term prognosis in patients undergoing rectal cancer surgery without neoadjuvant therapy
Source: World J Surg Oncol. 2022 Feb 25;20:51. doi: 10.1186/s12957-021-02460-7 (PMC8881874; doi:10.1186/s12957-021-02460-7)

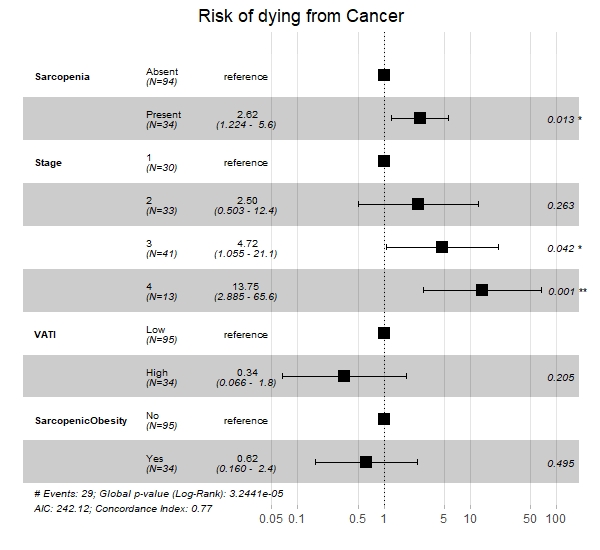

Supplement: Supplementary file 2 — Additional file 2: Supplementary Fig. 1 Multivariate analysis of factors associated to cancer-related death in rectal cancer patients undergoing upfront surgery. Quadrangles represent hazard risk. Horizontal bars represent 95% confidential interval VATI: Visceral adipose tissue index. [file 12957_2021_2460_MOESM2_ESM.jpeg]

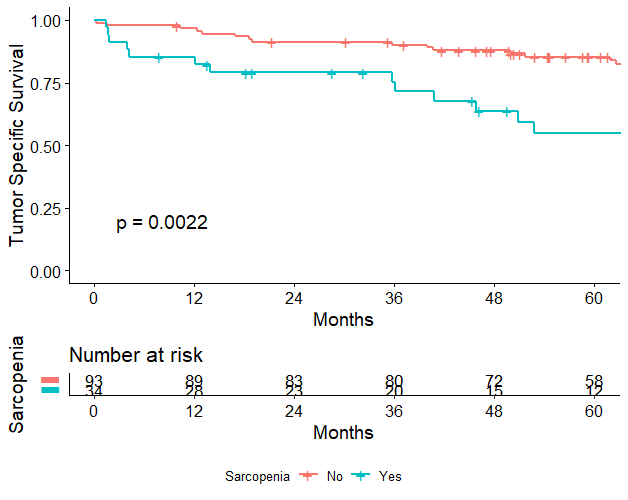

Supplement: Supplementary file 3 — Additional file 3: Supplementary Fig. 2 Kaplan-Meier tumor-specific survival curve for sarcopenic and non sarcopenic rectal cancer patients. [file 12957_2021_2460_MOESM3_ESM.png]

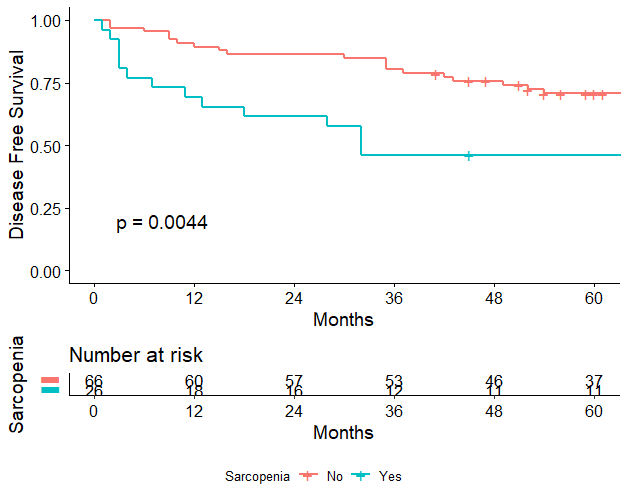

Supplement: Supplementary file 4 — Additional file 4: Supplementary Fig. 3 Kaplan-Meier disease-free survival curve for sarcopenic and non sarcopenic rectal cancer patients. [file 12957_2021_2460_MOESM4_ESM.png]
